# Sarkopenie, körperliche Aktivität und sedentäres Verhalten von Pflegeheimbewohnenden in Deutschland

**DOI:** 10.1007/s00391-023-02275-z

**Published:** 2024-01-26

**Authors:** Daniel Haigis, Silas Wagner, Rebekka Pomiersky, Leon Matting, Lea-Sofie Hahn, Gerhard W. Eschweiler, Ansgar Thiel, Annika Frahsa, Gorden Sudeck, Andreas M. Nieß

**Affiliations:** 1 Abteilung Sportmedizin, Universitätsklinikum Tübingen, 72076 Tübingen, Deutschland; 2 Interfakultäres Forschungsinstitut für Sport und körperliche Aktivität, Eberhard Karls Universität Tübingen, 72074 Tübingen, Deutschland; 3 Institut für Sportwissenschaft, Eberhard Karls Universität Tübingen, 72074 Tübingen, Deutschland; 4 Geriatrisches Zentrum, Universitätsklinikum Tübingen, 72076 Tübingen, Deutschland; 5 Institut für Sozial- und Präventivmedizin, Universität Bern, 3012 Bern, Schweiz

**Keywords:** Akzelerometrie, Bewegungsförderung, EWGSOP2, Pflegeheim, Prävention, Accelerometry, Physical acivity promotion, EWGSOP2, Nursing home, Prevention

## Abstract

**Hintergrund:**

Bewohnende in Pflegeheimen (PH) weisen eine hohe Prävalenz des muskuloskeletalen Syndroms Sarkopenie auf und erreichen häufig nicht die aktuellen Empfehlungen für körperliche Aktivität (kA).

**Fragestellung:**

Ziel dieser Studie ist die Identifizierung der Unterschiede in Bezug auf kA und sedentäres Verhalten (sV) von sarkopenen Bewohnenden im Vergleich zu nichtsarkopenen und präsarkopenen Bewohnenden.

**Methoden:**

Die Sarkopeniequantifizierung wurde bei 63 Bewohnenden aus PH in Baden-Württemberg (DE) anhand der Vorgaben der *European Working Group on Sarcopenia in Older People 2* durchgeführt. Untersucht wurden strukturierte Aktivitätseinheiten (sAE/Woche), akzelerometrisch erfasste kA (Schritte/Tag) und prozentuales sV (sV%/Tag). Die Gruppenvergleiche wurden mithilfe des Kruskal-Wallis-Tests und des Dunn-Bonferroni-Post-hoc-Tests ermittelt.

**Ergebnisse:**

Signifikante Unterschiede zeigten sich für Schritte (*p* = 0,005) und sV% (*p* = 0,019). Darüber hinaus zeigten die Schritte signifikante Ergebnisse im Vergleich der Gruppen „keine Sarkopenie“ (2824,4 [423–14275]) und „mögliche Sarkopenie“ (1703,9 [118–5663]) bzw. „bestätigte/schwere Sarkopenie“ (1571,2 [240–2392]) (beide *p* = 0,022; |r| = 0,34). Das sV% zeigte signifikante Unterschiede zwischen den Gruppen „keine Sarkopenie“ (87,9 % [69,1–94,3]) und „mögliche Sarkopenie“ (91,7 % [80,4–98,5]) (*p* = 0,018; |r| = 0,35).

**Diskussion:**

Nichtsarkopene Bewohnende weisen im Vergleich zu präsarkopenen und sarkopenen Bewohnenden eine höhere Schrittzahl und niedrigeres sV% auf. Die Erhöhung der Schrittzahl, die Verringerung des sV% und Förderung von Alltagsaktivitäten können für die Prävention und Behandlung der Sarkopenie im Setting PH beitragen.

**Zusatzmaterial online:**

Zusätzliche Informationen sind in der Online-Version dieses Artikels (10.1007/s00391-023-02275-z) enthalten.

## Abstract (en)

**Background:**

Residents in nursing homes show a high prevalence of the musculoskeletal syndrome sarcopenia and tend not to achieve current physical activity recommendations.

**Objective:**

The aim of this study is to identify differences in physical activity and sedentary behavior of sarcopenic residents compared with nonsarcopenic and presarcopenic residents.

**Methods:**

Sarcopenia assessment was performed among 63 nursing home residents in Baden-Wuerttemberg (D) using the *European Working Group on Sarcopenia in Older People 2* specifications. Structured activity sessions (per week), accelerometer-based physical activity (steps/day), and sedentary behavior (percentual/day) were examined. The group comparisons were determined with Kruskal-Wallis tests and Dunn-Bonferroni post hoc tests.

**Results:**

Significant differences were found for number of steps (*p* = 0.005) and percentual sedentary behavior (*p* = 0.019). Moreover, steps per day presented significant results in group comparison for no sarcopenia (2824.4 [423–14275]) with probable sarcopenia (1703.9 [118–5663]) and confirmed/severe sarcopenia (1571.2 [240–2392]) (both *p* = 0.022; |r| = 0.34). Sedentary behavior demonstrated significant differences in groups with no sarcopenia (87.9% [69.1–94.3]) and with probable sarcopenia (91.7% [80.4–9835]) (*p* = 0.018; |r| = 0.35).

**Conclusion:**

Nonsarcopenic residents demonstrated a higher number of steps and lower sedentary behavior compared with presarcopenic and sarcopenic residents. Increasing steps, reducing sedentary behavior and promoting activities of daily living can contribute to the prevention and treatment of sarcopenia in the nursing home setting.

## Hinführung zum Thema

Die Prävention und Behandlung der Sarkopenie sind im Setting Pflegeheim (PH) aufgrund von schweren körperlichen und geistigen Beeinträchtigungen der Bewohnenden erschwert, wobei nationale und internationale Bewegungsempfehlungen kaum umsetzbar sind. Bisher gibt es jedoch keine settingspezifischen Studien in Deutschland, welche die Sarkopenie im Kontext der körperlichen Aktivität (kA) und sedentäres Verhalten (sV) betrachteten. Demnach soll diese Studie mit ihren Ergebnissen zur Verbesserung der Bewegungsförderung im Setting PH beitragen.

## Hintergrund

Die Sarkopenie ist ein Syndrom, welches durch den Verlust von Muskelkraft in Kombination mit dem Verlust von Muskelmasse und Muskelfunktion beschrieben wird [1]. Sie ist mit einem erhöhten Sturzrisiko sowie dem Verlust der Mobilität und Selbstständigkeit verbunden. Darüber hinaus beeinträchtigt die Sarkopenie nicht nur die körperliche Gesundheit, sondern beeinflusst auch die soziale Teilhabe der Betroffenen [2]. Nicht zuletzt gilt die Sarkopenie als Prädiktor für die Mortalität in PH [3]. Hier liegt der Anteil sarkopener Bewohnenden zwischen 17,7 und 87,0 % [4]. Sie sind besonders auf die aktuellen Empfehlungen für die Diagnose und Behandlung der Sarkopenie angewiesen. Allerdings werden bisherige Forschungskenntnisse nur unzureichend in das Setting PH übertragen. Hauptsächlich bestehen Schwierigkeiten der Umsetzung in die Praxis bei der Quantifizierung, Prävention und Behandlung [5].

Nationale und internationale Empfehlungen zur kA weisen auf die Notwendigkeit der Förderung von älteren Personen hin [6, 7]. Neben Krafttraining in Kombination mit Gleichgewichts- und Ausdauertraining ist kA eine wirksame Form der Sarkopenieprävention und -behandlung, da sie die Muskelmasse, Muskelkraft und körperliche Funktionsfähigkeit maßgeblich beeinflusst. Ein wichtiger Punkt der Empfehlungen ist die Steigerung der allgemeinen kA, die so oft wie möglich in den Alltag integriert werden sollte [8]. Frühere Studien haben jedoch gezeigt, dass sich Bewohnende in PH zu wenig bewegen.

Bisher wurden in keinen Studien die Sarkopenie bei Bewohnenden in deutschen PH im Kontext der kA und des sV untersucht. Daher stellt sich die Frage, welche Unterschiede es zwischen Bewohnenden von PH in Bezug auf den Sarkopeniestatus und deren kA und sV gibt, Diese Frage ist von entscheidender Bedeutung, um Möglichkeiten und Barrieren für die Prävention und Behandlung der Sarkopenie sowie künftige Empfehlungen zur kA für Bewohnende in PH zu identifizieren. Ziel unserer Studie ist es daher, den Sarkopeniestatus der Bewohnenden von PH gemäß den Leitlinien der *European Working Group on Sarcopenia in Older People 2* (EWGSOP2; [1]) zu quantifizieren. Darüber hinaus werden die Bewohnenden in Abhängigkeit ihrer strukturierten Aktivitätseinheiten (sAE), kA und sV untersucht, um Gruppenvergleiche zu ermöglichen. Die Ergebnisse sollen die Notwendigkeit einer settingspezifischen Bewegungsförderung unterstützen.

## Methoden

### Rekrutierung, Ein- und Ausschlusskriterien

Diese Untersuchung war Teil des BaSAlt-Projekts, welches das Ziel der Bewegungsförderung in deutschen PH verfolgte [9]. Das BaSAlt-Projekt wurde durch das Bundesministerium für Gesundheit gefördert (Projektnummer ZMVI1-2519FSB114; Förderzeitraum Juni 2019–Juni 2023). Die Baseline-Erhebung wurde zwischen September 2020 und April 2022 in 5 PH in Baden-Württemberg (DE) durchgeführt. Vor den Assessments wurden 11 Assessoren aus den kooperierenden PH in einem zweitägigen Workshop durch das BaSAlt-Team für die Assessments geschult. Einschlusskriterium für die Studie war ein Pflegegrad ≤ 4 (Einstufung der Pflegegrade 1–5). Ausschlusskriterien waren/war ein Pflegegrad 5 und/oder ein palliativer Zustand mit schweren körperlichen oder geistigen Beeinträchtigungen sowie Bettlägerigkeit.

Die BaSAlt-Studie wurde unter Berücksichtigung der ethischen Anforderungen der Deklaration von Helsinki und Ethikkommission der Wirtschafts- und Sozialwissenschaftlichen Fakultät der Eberhard Karls Universität Tübingen (Ethikvotum Nr. AZ A2.5.4-096_aa; Einreichung Juli 2019) durchgeführt. Die schriftliche Einwilligung zur Studienteilnahme wurde von den Bewohnenden und/oder einer gesetzlichen Vertretung eingeholt.

### Demografische und anthropometrische Daten

Das Geschlecht, Alter (in Jahren), Größe (in m), Gewicht (in kg), Body-Mass-Index (BMI in kg/m^2^) und Pflegegrad (1–4) der Bewohnenden wurden erfasst.

Die Kategorisierung des Morbiditätsstatus in früheres kardiovaskuläres Ereignis, arterielle Hypertonie, koronare Herzkrankheit, Herzinsuffizienz; Herzschrittmacher, Schlaganfall/Hirnblutung/TIA, chronische Lungenerkrankung, Krebserkrankung, Diabetes mellitus Typ 2, Arthrose der unteren Extremität, psychische/emotionale/nervale Erkrankung erfolgte durch genehmigte Einsicht in die Bewohnendenakte.

Die kognitive Funktion wurde mittels Mini-Mental-Status-Test (MMST) bewertet. Maximal 30 Punkte konnten erreicht werden und bedeuteten eine uneingeschränkte kognitive Funktion [10].

Die Pflegebedürftigkeit wurde mit dem Barthel-Index (BI) ermittelt. In 10 Einzelkategorien des täglichen Lebens konnten insgesamt 100 Punkte erreicht werden. Eine maximale Punktzahl bedeutete eine uneingeschränkte Alltagsfunktion [11].

Um das Sarkopenierisiko zu ermitteln, erfolgte die Erhebung anhand des SARC-F-Fragebogens. Der SARC‑F besteht aus 5 Fragen zur körperlichen Funktionsfähigkeit. Mithilfe eines Punktesystems können insgesamt 10 Punkte erreicht werden. Wurden ≥4 Punkte erreicht, bestand der Verdacht einer Sarkopenie [12]. Für die Sarkopeniequantifizierung nach EWGSOP2-Vorgaben wurde der SARC‑F in dieser Studie nicht berücksichtigt, da eine eingeschränkte Selbstauskunft der Bewohnenden aufgrund kognitiver Veränderungen zu erwarten war.

### Sarkopeniequantifizierung

Untersuchungen zur Bestimmung der Sarkopenie beinhalteten die Muskelkraft durch Messung der maximalen Handkraft (mHK in kg) mit einem Handkraftdynamometer (Fa. Saehan, Model SH5001, Changwonsi, Korea). Drei Messungen wurden abwechselnd mit der rechten und linken Hand durchgeführt. Der beste Wert aus 6 Messungen wurde berücksichtigt [13]. Wurde der geschlechterspezifische Cut-off-Wert der mHK unterschritten, wurde eine „möglichen Sarkopenie“ identifiziert.

Um den Verdacht der Sarkopenie zu bestätigen, wurde die Muskelmasse bestimmt. Die appendikuläre Skelettmuskelmasse (aSMM in kg) wurde mittels dem Bioimpedanzanalysegerät (Fa. Akern, BIA 101 BIVA (50 kHz ± 1 % Messfrequenz), Pontassieve, Italien) gemessen und mit der BodygramPlus Enterprise Software Version 1.2.2.9, Akern s.r.l. ausgewertet [14]. Lag der aSMM unter den geschlechtsspezifischen Cut-off-Werten, erfolgte die Quantifizierung der Bewohnenden in die Gruppe „bestätigte Sarkopenie“.

Der Schweregrad der Sarkopenie definierte sich anhand der körperlichen Funktionsfähigkeit der Bewohnenden. Die weitere Quantifizierung bestand aus der Messung der habituellen 4‑Meter-Ganggeschwindigkeit (4mGG in m/s). Die Messung erfolgte normiert über eine Strecke von 4 m. Eine An- und Auslaufstrecke von jeweils 2 m wurde zusätzlich berücksichtigt [15]. Bewohnende, die nicht selbstständig gehen konnten, wurden ebenso als eingeschränkt in ihrer körperlichen Funktionsfähigkeit definiert, wie Bewohnende, welche die Cut-off-Werte unterschritten. Die Sarkopeniequantifizierung erfolgte demnach in die Gruppe „schwere Sarkopenie“.

### Körperliche Aktivität und sedentäres Verhalten

Für die Dokumentation der sAE wurde ein standardisierter Fragebogen zur Befragung der Bewohnenden und/oder Assessoren*Innen verwendet. Betreute Gruppenaktivitäten, die von Mitarbeitenden der PH organisiert wurden, sowie betreute Einzelaktivitäten durch Physiotherapeuten*Innen und individuelle Aktivitäten mit Betreuungskräften und Angehörigen wurden erfasst. Die Teilnahme und Häufigkeit pro Woche wurden ermittelt.

Die kA und sV wurden mit einem ActiGraph wGT3x-BT (Fa. ActiGraph, Pensacola, FL, USA) gemessen. Die Aktivitätsdaten wurden an 7 aufeinanderfolgenden Tagen, einschließlich Wochentagen und Wochenendtagen, erfasst. Die Rohdaten wurden mit einer Abtastrate von 30 Hz und einer Epochenlänge von 10 s ausgewählt. Nur Personen mit einer gültigen Tragedauer (mindestens 10-h-Tragedauer zwischen 6 Uhr morgens und 22 Uhr abends pro Tag) an mindestens 3 gültigen Tagen wurden in die weitere Analyse einbezogen [16]. Die Werte kA in Schritten/Tag und prozentuales sV wurden mit der Software ActiLife 6 berechnet [17, 18].

### Statistische Analyse

Für die deskriptive Analyse wurden Medianwerte (Md) und Bereiche (Minimum bis Maximum) berechnet. Für den Gruppenvergleich der Sarkopeniegruppen wurde das nichtparametrische Verfahren mittels Kruskal-Wallis-Test verwendet. Es wurde ein Dunn-Bonferroni-Post-hoc-Test für den Vergleich zwischen den Sarkopeniegruppen mit den Variablen Schritte und sV% berechnet. Das Signifikanzniveau wurde auf *p* ≤ 0,05 festgelegt (2-seitige Testung). Es wurden Effektgrößen |r| nach Cohen berechnet und als kleiner (|r| ≥ 0,10), moderater (|r| ≥ 0,30) und großer (|r| ≥ 0,50) Effekt interpretiert.

## Ergebnisse

**Abb. 1:**
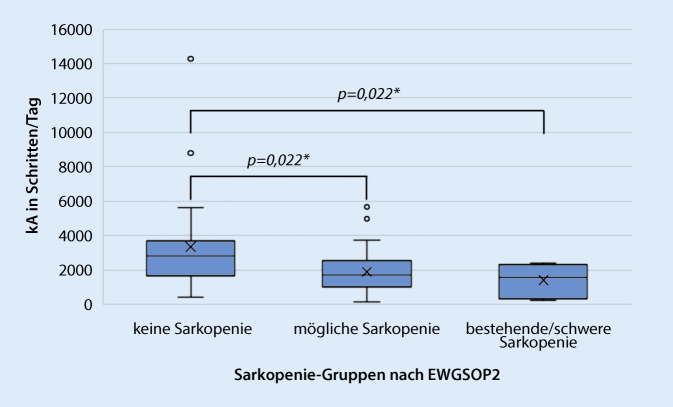
Boxplot für körperliche Aktivität in Schritten/Tag. (Boxplots mit Mittelwert und Interquartilsabstand [Q1–Q3]; Median [*X*] gekennzeichnet mit Minimum bis Maximum [*Antennen*], Ausreißer > 1,5fach des Interquartilsabstands. *kA* körperliche Aktivität, *EWGSOP2* European Working Group on Sarcopenia in Older People 2. *signifikante Ergebnisse des Dunn-Bonferroni-Post-hoc-Tests *p* ≤ 0,05)

Insgesamt wurden 81 Bewohnende aus 5 PH rekrutiert. Davon konnten letztlich 63 Bewohnende für die Analyse berücksichtigt werden. Ausgeschlossen wurden Bewohnende, die zur Baseline-Erhebung das Heim verlassen haben (*n* = 1), sich im palliativen Zustand befanden (*n* = 1) oder verstorben waren (*n* = 1). Die vorausgesetzte Tragezeit des Akzelerometers war zudem bei 15 Bewohnenden nicht gültig. Fehlende Werte und deren Gründe wurden für alle Variablen beschrieben und in der Analyse nicht berücksichtigt (Zusatzmaterial online: Appendix 1). Nur ein Bewohnender wurde in die Gruppe „bestätigte Sarkopenie“ eingestuft. Daher wurden die Sarkopeniegruppen „bestätigte Sarkopenie“ und „schwere Sarkopenie“ für die weitere Analyse zusammengefasst. Tab. 1 zeigt die deskriptive Analyse und Gruppenvergleiche anhand der Kruskal-Wallis-Tests. Der Pflegegrad lag in allen 3 Gruppen im Mittel bei 3 (2–4). Weitere detaillierte deskriptive Daten werden im Zusatzmaterial online: Appendix 2 und 3 beschrieben. Für alle Arten von sAE wurden keine signifikanten Unterschiede im Gruppenvergleich bestimmt (*p* ≥ 0,05). Tab. 2 zeigt die Analyse durch Dunn-Bonferroni-Post-hoc-Tests für den Gruppenvergleich für Schritte und sV%. Zusätzlich sind Boxplots in den Abb. 1 und 2 für die Variablen Schritte und sV% dargestellt.

**Tab. 1:** Deskriptive Statistik und Gruppenvergleiche nach Kruskal-Wallis-Test

	Keine Sarkopenie	Mögliche Sarkopenie	Bestätigte/schwere Sarkopenie	Kruskal-Wallis‑H *p*-Wert
	*n*_ges_ = 27 (42,9 %)	*n*_gesl_ = 26 (41,2 %)	*n*_ges_ = 10 (15,9 %)	
	*n*_w_ = 21 (77,8 %)	*n*_w_ = 17 (65,4 %)	*n*_w_ = 9 (90,0 %)	
	*n*_m_ = 6 (22,2 %)	*n*_m_ = 9 (34,6 %)	*n*_m_ = 1 (10,0 %)	
Alter in Jahren^a^	86,0(65–98)	86,0(64–94)	89,0(71–93)	H = 1,653
				*p* = 0,438
Größe in cm^a^	162,0(150–175)	166,5(144–187)	152,5(140–174)	H = 7,997
				*p* = 0,018*
Gewicht in kg^a^	68,8(41,4–101,0)	72,0(53,1–110,5)	56,2(36,4–70,2)	H = 12,904
				*p* = 0,002*
BMI in kg/m^2a^	25,1(17,5–43,1)	24,9(20,9–45,4)	23,4(15,5–27,5)	H = 8,275
				*p* = 0,016*
Morbiditätsstatus^a^	3,0(1–5)	4,0(1–10)	2,5(0–6)	H = 1,611
				*p* = 0,447
MMST in Punkte^a^	25,0(4–30)	19,5(2–30)	14,5(9–27)	H = 3,488
				*p* = 0,175
BI in Punkten^a^	85,0(25–100)	65,0(10–100)	57,5(25–75)	H = 14,674
				*p* < 0,001*
SARC‑F in Punkten^a^	3,0(0–9)	3,0(0–10)	4,0(0–7)	H = 0,897
				*p* = 0,639
*Bestehendes Sarkopenierisiko*	*n* = 9(33,3 %)	*n* = 7(26,9 %)	*n* = 6(60,0 %)	–
*Kein Sarkopenierisiko*	*n* = 16(59,3 %)	*n* = 15(57,7 %)	*n* = 2(20,0 %)	
mHK in kg^a^	18,0(16–40)	14,0(5–26)	11,5(3–26)	H = 21,217
				*p* < 0,001*
aSMM in kg^a^	16,1(11,5–25,7)	18,9(15,0–31,4)	13,0(8,4–18,1)	H = 20,020
				*p* < 0,001*
4mGG in m/s^a^	0,60(0,27–0,95)	0,59(0,32–1,02)	0,55(0,44–0,90)	H = 0,083
				*p* = 0,959
kA in Schritten/Tag^a^	2824,4(423–14275)	1703,9(118–5663)	1571,2(240–2392)	H = 14,495
				*p* = 0,005*
sV prozentual/Tag^a^	87,9 % (69,1–94,3)	91,7 % (80,4–98,5)	90,3 % (84,7–96,9)	H = 7,972
				*p* = 0,019*

*4mGG* 4-Meter-Ganggeschwindigkeit, *aSMM* appendikuläre Skelettmuskelmasse, *BI* Barthel-Index, *BMI* Body-Mass-Index, *kA* körperliche Aktivität, *MMST* Mini-Mental-Status-Test, *mHK* maximale Handkraft, *sV* sedentäres Verhalten

^a^Medianwert (Minimum bis Maximum)

*signifikante Ergebnisse *p* ≤ 0,05

**Tab. 2:** Dunn-Bonferroni-Post-hoc-Tests für körperliche Aktivität und sedentäres Verhalten

	z‑Wert	Effektstärke	*p*-Wert
*kA in Schritten/Tag*			
Keine Sarkopenie – mögliche Sarkopenie	z = 2,677	|r| = 0,34	*p* *=* 0,022*
Keine Sarkopenie – bestätigte/schwere Sarkopenie	z = 2,674	|r| = 0,34	*p* *=* 0,022*
Mögliche Sarkopenie – bestätigte/schwere Sarkopenie	z = 0,683	|r| = 0,09	*p* *≥* *0,999*
*sV prozentual/Tag*			
Keine Sarkopenie – mögliche Sarkopenie	z = −2,746	|r| = 0,35	*p* *=* 0,018*
Keine Sarkopenie – bestätigte/schwere Sarkopenie	z = −1,613	|r| = 0,20	*p* *=* *0,320*
Mögliche Sarkopenie – bestätigte/schwere Sarkopenie	z = 0,423	|r| = 0,05	*p* *≥* *0,999*

*kA* körperliche Aktivität, *sV* sedentäres Verhalten

*signifikante Ergebnisse *p* ≤ 0,05

**Abb. 2:**
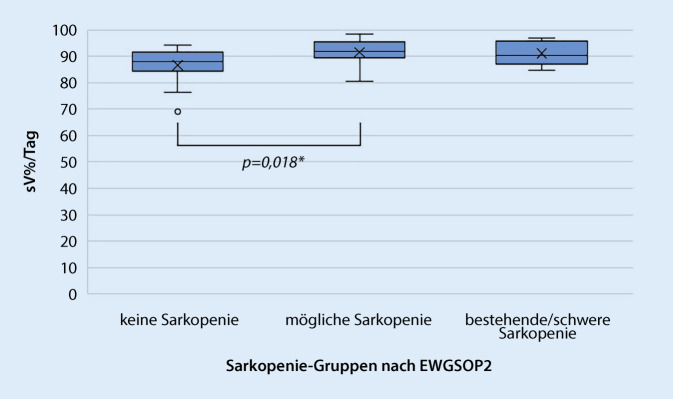
Boxplot für sedentäres Verhalten prozentual/Tag. (Boxplots mit Mittelwert und Interquartilsabstand [Q1–Q3]; Median [*X*] gekennzeichnet mit Minimum bis Maximum [*Antennen*], Ausreißer > 1,5fach des Interquartilsabstands. *sV* sedentäres Verhalten, *EWGSOP2* European Working Group on Sarcopenia in Older People 2. *signifikante Ergebnisse des Dunn-Bonferroni-Post-hoc-Tests *p* ≤ 0,05)

## Diskussion

Das Ziel dieser Studie war die Untersuchung von Bewohnenden in PH auf ihren Sarkopeniestatus, kA und sV. Nach unserem Kenntnisstand ist dies die erste Studie, die sich mit den Unterschieden zwischen kA und sV bei Bewohnenden aus deutschen PH in Bezug auf die Sarkopenie befasste. Im internationalen Vergleich konnte gezeigt werden, dass in der BaSAlt-Kohorte die Prävalenz der Sarkopenie (15,9 %) vergleichsweise gering ausfiel, da bettlägerige Bewohnende ausgeschlossen wurden. Dennoch wies ein Großteil der Bewohnenden einen präsarkopenen Status auf (41,2 %). Gemäß den EWGSOP2-Leitlinien weist dieser präsarkopene Zustand auf die Notwendigkeit einer flächendeckenden Intervention hin, um das Fortschreiten der Sarkopenie zu verhindern [1].

Die Ergebnisse zeigen signifikante Unterschiede der kA und sV zwischen nichtsarkopenen und präsarkopenen Bewohnenden und die Notwendigkeit, der Sarkopenie so früh wie möglich präventiv entgegenzuwirken. Es kann davon ausgegangen werden, dass insbesondere ältere Personen mit einem hohen Risiko für die Entwicklung einer Sarkopenie eine individuelle Trainingsintervention benötigen. Dementsprechend sollten auch körperlich und kognitiv *fittere* Bewohnende durch kA in PH gefördert werden. Eine entsprechende Förderung der kA sollte bereits bei der Aufnahme der Bewohnenden in das PH durchgeführt werden. Die Anpassung an die individuellen Bedürfnisse sollten dabei fokussiert werden, um der Sarkopenie vorzubeugen. Die kA und individuell zugeschnittene Bewegungsprogramme zeigen signifikante Effekte auf Muskelmasse, Muskelkraft und körperliche Funktionsfähigkeit. Diesen Sarkopeniekomponenten könnte durch Bewegungsförderung und -maßnahmen gezielt entgegengewirkt werden [19]. Die aktuellen Empfehlungen für kA bauen auf verschiedenen Altersgruppen auf und definieren deren Empfehlungen für ältere Personen > 65 Jahre mit ≥ 150 min/Woche moderater und/oder ≥ 75 min/Woche intensiver kA, wobei zusätzliche Kraft- und Gleichgewichtsübungen integriert werden sollen [6, 7]. Die Empfehlungen sind jedoch oft komplex und nicht spezifiziert, was für pflegebedürftige und kognitiv beeinträchtigte Bewohnende in PH deren Durchführung erschwert. In der deutschen Bevölkerung > 70 Jahren liegt die Compliance mit den Empfehlungen zur kA bei 65,6 % [20]. Mit zunehmendem Alter kommt es jedoch zu einer signifikanten Verringerung der kA und zu einer Zunahme des sV [21]. Dies spiegelt sich deutlich in unseren Studienergebnissen wider, da in dem Setting PH eine hohe Anzahl an immobilen Bewohnenden vorzufinden ist. Auch weisen die signifikanten Gruppenunterschiede von BMI, BI und mHK auf weitere Einflussfaktoren für das verringerte Aktivitätsniveau der Bewohnenden hin. Wider Erwarten bestehen jedoch keine signifikanten Gruppenunterschiede in Bezug auf die 4mGG, was die allgemeine körperliche Funktionseinschränkung von Bewohnenden im Setting PH darstellt.

Darüber hinaus zeigte unsere Studie, dass sich die Art der kA zwischen den beiden Sarkopeniegruppen nicht signifikant unterscheidet. Einen entscheidenden Faktor stellt demnach die Maximierung der kA bzw. Alltagsaktivität dar. Ein besonderes Augenmerk sollte deshalb auf die körperliche Funktionsfähigkeit gelegt werden, da diese eine Voraussetzung für kA darstellt. Dies zeigt sich in unserer Studie, da der Anteil der Bewohnenden mit körperlichen Funktionseinschränkungen überwiegt. Dies trägt auch zur Prävalenz der schweren Sarkopenie in der BaSAlt-Kohorte bei. Um jedoch konkrete Empfehlungen für eine Bewegungsförderung im Setting PH auszusprechen, werden hierfür weitere Untersuchungen und zusätzlich evidente Interventionsmaßmaßnehmen zur interdisziplinären Prävention und Behandlung der Sarkopenie im Setting PH im Längsschnitt benötigt.

## Stärken und Limitationen

Die Objektivierung der körperlichen (In‑)Aktivität mittels Akzelerometrie sowie die Unterstützung durch geschulte Assessoren konnten die Datenqualität im Vergleich zu bisherigen Studien verbessern.

Limitationen zeigen sich an der geringen Teilnehmendenzahl mit einer Vielzahl an Multimorbiditäten, dem Vorliegen von Querschnittsdaten sowie den Einschränkungen der Bewegungsangebote, sozialen Aktivitäten und Kontaktverboten durch die Coronapandemie zum Zeitpunkt der Datenerhebung in PH.

## Fazit für die Praxis

- Zunehmendes Alter geht mit sinkender körperlichen Aktivität, zunehmendem sedentären Verhalten und Sarkopenieprävalenz einher, was eine zukünftige Herausforderung für das Gesundheitssystem darstellen wird.
- Hohe Anzahl an präsarkopenen Bewohnenden bietet Chancen für Sarkopenieprävention im Setting Pflegeheim.
- Zur Sarkopenieprävention sollten Maßnahmen der Bewegungsförderung auf organisationaler und individueller Ebene erfolgen.
- Berücksichtigung individueller Bedürfnisse und ein standardisiertes Sarkopenieassessment können Bewegungsförderung im Setting Pflegeheim gezielt unterstützen.

### Supplementary Information

Zusätzliche Informationen zu fehlenden Werten und deren Gründe wurden für alle Variablen in Appendix 1 beschrieben. Zusätzlich werden weitere detaillierte deskriptive Daten, bspw. zu Morbiditätskategorien, in Appendix 2 und 3 beschrieben.
