# Supplementary material for: Sarcopenia, physical activity and sedentary behavior among nursing home residents in Germany
Source: Z Gerontol Geriatr. 2024 Jan 26;57(5):395–401. [Article in German] doi: 10.1007/s00391-023-02275-z (PMC11315749; doi:10.1007/s00391-023-02275-z)
Supplement: Supplementary file 1 — Zusätzliche Informationen zu fehlenden Werten und deren Gründe wurden für alle Variablen in Appendix 1 beschrieben. Zusätzlich werden weitere detaillierte deskriptive Daten, bspw. zu Morbiditätskategorien, in Appendix 2 und 3 beschrieben. [file 391_2023_2275_MOESM1_ESM.docx]

Appendix 1. Fehlende Werte mit Begründungen.

|  | Anzahl fehlende Werte | Gründe |
| --- | --- | --- |
| Morbiditätsstatus | n= 1 | Keine Einwilligung Einsicht Bewohnendenakte (n=1) |
| MMST | n=7 | stark eingeschränkte Sehkraft (n=4) mangelnde Motivation (n=2) starke kognitive Beeinträchtigung (n=1) |
| SARC-F | n=8 | schwere kognitive Beeinträchtigungen (n=7) mangelnde Motivation (n=1) |
| mHK | n=2 | schwerer kognitiver Beeinträchtigungen (n=2) |
| 4mGG | n=6 | keine selbstständige Fortbewegung möglich (n=6) |
| sAE | n=1 | keine Werte vorliegend (n=1) |

*4mGG: 4-Meter-Ganggeschwindigkeit; MMST: Mini-Mental-Status-Test; mHK: maximale Handkraft; sAE: strukturierte Aktivitätseinheiten.*

Appendix2. Kategorisierung von Pflegegrad, Morbiditäten, Mini-Mental-Status-Test und Barthel-Index.

|  | keine Sarkopenie | mögliche Sarkopenie | bestätigte/schwere Sarkopenie |
| --- | --- | --- | --- |
| Pflegegrad^†^ | 3,0(2-4) | 3,0(2-4) | 3,0(2-4) |
| *2* | n=10(37,0%) | n=6(23,1%) | n=2(20,0%) |
| *3* | n=13(48,2%) | n=15(57,7%) | n=6(60,0%) |
| *4* | n=4(14,8%) | n=5(19,2%) | n=2(20,0%) |
| Morbiditätsstatus^†^ | 3,0(1-5) | 4,0(1-10) | 2,5(0-6) |
| *früheres kardiovaskuläre Ereignis* | n=7(25,9%) | n=7(26,9%) | n=3(30,0%) |
| *arterielle Hypertonie* | n=17(63,0%) | n=19(73,1%) | n=5(50,0%) |
| *koronare Herzkrankheit* | n=4(14,8%) | n=10(38,5%) | n=2(20,0%) |
| *Herzinsuffizienz* | n=8(29,6%) | n=9(34,6%) | n=3(30,0%) |
| *Herzschrittmacher* | n=3(11,1%) | n=3(11,5%) | n=1(10,0%) |
| *Schlaganfall/Hirnblutung/TIA* | n=6(22,2%) | n=5(19,2%) | n=2(20,0%) |
| *chronische Lungenerkrankung* | n=0(0,0%) | n=3(11,5%) | n=1(10,0%) |
| *Krebserkrankung* | n=5(18,5%) | n=5(19,2%) | n=1(10,0%) |
| *Diabetes mellitus II* | n=7(25,9%) | n=10(38,5%) | n=2(20,0%) |
| *Arthrose untere Extremität* | n=9(33,3%) | n=10(38,5%) | n=3(30,0%) |
| *psychische/emotionale/nervale Erkrankung* | n=17(63,0%) | n=12(46,2%) | n=7(70,0%) |
| MMST in Punkte^†^ | 25,0(4-30) | 19,5(2-30) | 14,5(9-27) |
| *keine Demenz* | n=7(25,9%) | n=4(15,4%) | n=0(0,0%) |
| *leicht kognitive Einschränkung* | n=6(22,2%) | n=5(19,2%) | n=2(20,0%) |
| *leichte Demenz* | n=6(22,2%) | n=4(15,4%) | n=1(10,0%) |
| *moderate Demenz* | n=5(18,5%) | n=10(38,5%) | n=4(40,0%) |
| *schwere Demenz* | n=1(3,7%) | n=1(3,8%) | n=1(10,0%) |
| BI in Punkte^†^ | 85,0(25-100) | 65,0(10-100) | 57,5(25-75) |
| *nicht pflegebedürftig* | n=2(7,4%) | n=1(3,8%) | n=0(0,0%) |
| *teilweise pflegebedürftig* | n=13(48,1%) | n=6(23,1%) | n=0(0,0%) |
| *pflegebedürftig* | n=11(40,7%) | n=17(65,4%) | n=9(90,0%) |
| *vollständig pflegebedürftig* | n=1(3,7%) | n=2(7,7%) | n=1(10,0%) |

4mGG: 4-Meter-Ganggeschwindigkeit; aSMM: appendikuläre Skelettmuskelmasse; BI: Barthel-Index; BMI: Body-Mass-Index; MMST: Mini-Mental-Status-Test; mHK: maximale Handkraft; **^†^**Medianwert (Minimum-Maximum).

Appendix 3. Kategorisierung strukturierte Aktivitätseinheiten.

|  | keine Sarkopenie | mögliche Sarkopenie | bestätigte/schwere Sarkopenie |
| --- | --- | --- | --- |
| angeleitete Gruppenaktivitäten | | | |
| Teilnahme | n=23(85,2%) | n=22(84,6%) | n=6(60,0%) |
| Anzahl/Woche^†^ | 1,5(0-4) | 2,0(0-4) | 2,0(0-4) |
| angeleitete individuelle Aktivitäten (Physiotherapie) | | | |
| Teilnahme | n=11(40,7%) | n=13(50,0%) | n=3(30,0%) |
| Anzahl/Woche^†^ | 0,0(0-2) | 0,0(0-4) | 0,0(0-3) |
| individuelle Aktivitäten (Besuchsdienst) | | | |
| Teilnahme | n=2(7,4%) | n=2(7,7%) | n=1(10,0%) |
| Anzahl/Woche^†^ | 0,0(0-8) | 0,0(0-1) | 0,0(0-4) |
| individuelle Aktivitäten (Angehörige) | | | |
| Teilnahme | n=23(85,2%) | n=21(80,8%) | n=8(80,0%) |
| Anzahl/Woche^†^ | 1.0(0-7) | 1.0(0-5) | 0.5(0-2) |

**^†^***Medianwert (Minimum-Maximum).*
